# First-Line Treatment After Perioperative FLOT in Recurrent Gastric and Gastroesophageal Junction Cancer: A Turkish Oncology Group (TOG) Multicenter Real-World Analysis

**DOI:** 10.3390/medicina62050984

**Published:** 2026-05-18

**Authors:** Mustafa Seyyar, Pervin Can Şancı, Abdullah Sakin, Ayberk Bayramgil, Özgecan Dülgar Kaya, Erdem Sünger, Özgür Açıkgöz, Teyfik Demir, Recep Türkel, Bahiddin Yılmaz, Faruk Recep Özalp, Hüseyin Salih Semiz, Gül Sema Yıldıran, Musa Barış Aykan, Görkem Turhan, Atila Yıldırım, Serkan Menekşe, Engin Kut, Mehmet Çakmak, Efnan Algın, Elif Şahin, Anıl Karakayalı, Aysel Oğuz, Mehmet Artaç, Mehmet Cihan İçli, Burak Paçacı, Murat Sarı, Teoman Şakalar, Murad Guliyev, Nebi Serkan Demirci, Eyyüp Çavdar, Ömer Faruk Elçiçek, Ali İnal, Hatice Bölek, Pınar Kubilay Tolunay, Ali Kalem, Melike Yazıcı, Ayşegül Merç Çetinkaya, Sinem Akbaş, Sedat Biter, Sait Kitaplı, Merve Kuday Özkan, Lamia Şeker Can, Nargiz Majidova, Hacı Arak, Hasan Çağrı Yıldırım, Devrim Çabuk, Kazım Uygun, Sema Sezgin Göksu, Özgür Tanrıverdi, Fatih Selçukbiricik, Mehmet Uzun, İlker Nihat Ökten, Burak Mete, Tolga Köşeci, Ahmet Bilici, Tülay Kuş, Ömer Dizdar, Şuayib Yalçın, Umut Kefeli

**Affiliations:** 1 Department of Medical Oncology, Gaziantep City Hospital, İbn-i Sina Neighborhood, 27470 Gaziantep, Türkiye; harak63@hotmail.com; 2 Department of Medical Oncology, Faculty of Medicine, Kocaeli University, 41380 Kocaeli, Türkiye; pervin.cansanci@saglik.gov.tr (P.C.Ş.); drmelikeyzc@gmail.com (M.Y.); devrim.cabuk@kocaeli.edu.tr (D.Ç.); kazim.uygun@kocaeli.edu.tr (K.U.); umut.kefeli@kocaeli.edu.tr (U.K.); 3 Department of Medical Oncology, Bahçelievler Medipol University Hospital, 34196 Istanbul, Türkiye; drsakin@hotmail.com; 4 Department of Medical Oncology, SBU Umraniye Training and Research Hospital, 34676 Istanbul, Türkiye; ayberkbayramgil@gmail.com (A.B.); ozgecandr@gmail.com (Ö.D.K.); 5 Department of Medical Oncology, Faculty of Medicine, Istanbul Medipol University, 34810 Istanbul, Türkiye; erdem.sunger@medipol.edu.tr (E.S.); ozgur.acikgoz@medipol.com.tr (Ö.A.); abilici@medipol.edu.tr (A.B.); 6 Department of Medical Oncology, Faculty of Medicine, Ondokuz Mayıs University, 55200 Samsun, Türkiye; teyfodemir462@gmail.com (T.D.); pecerturk@hotmail.com (R.T.); yilmazbahattin@hotmail.com (B.Y.); 7 Department of Medical Oncology, Faculty of Medicine, Dokuz Eylul University, 35340 Izmir, Türkiye; ozalpfarukrecep@gmail.com (F.R.Ö.); hsalihsemiz@hotmail.com (H.S.S.); 8 Department of Medical Oncology, Gulhane Training and Research Hospital, 06010 Ankara, Türkiye; gulsemayildiran@gmail.com (G.S.Y.); musabarisaykan@gmail.com (M.B.A.); 9 Department of Medical Oncology, Faculty of Medicine, Karadeniz Technical University, 61080 Trabzon, Türkiye; gorkemturhan@gmail.com (G.T.); dr_atila_yildirim@hotmail.com (A.Y.); 10 Department of Medical Oncology, Manisa City Hospital, 45010 Manisa, Türkiye; serkan.menekse@saglik.gov.tr (S.M.); drenginkut@gmail.com (E.K.); 11 Department of Medical Oncology, Ankara Bilkent City Hospital, 06800 Ankara, Türkiye; hcttpmehmet23@gmail.com (M.Ç.); efnanalgin@gmail.com (E.A.); hati.kocc@gmail.com (H.B.); 12 Department of Medical Oncology, Kocaeli City Hospital, 41060 Kocaeli, Türkiyeanil.karakayali@saglik.gov.tr (A.K.); 13 Department of Medical Oncology, Meram Faculty of Medicine, Necmettin Erbakan University, 42090 Konya, Türkiye; drayseloguz@gmail.com (A.O.); mehmetartac@yahoo.com (M.A.); 14 Department of Medical Oncology, Hacettepe University Cancer Institute, 06100 Ankara, Türkiye; mcihanic@gmail.com (M.C.İ.); dromerdizdar@gmail.com (Ö.D.); suayibyalcin@gmail.com (Ş.Y.); 15 Department of Medical Oncology, Marmara University Pendik Training and Research Hospital, 34899 Istanbul, Türkiye; drpacaci@gmail.com (B.P.); drmuratsari@gmail.com (M.S.); 16 Department of Medical Oncology, HG Hospital, 46050 Kahramanmaraş, Türkiye; drteomansakalar@gmail.com; 17 Department of Medical Oncology, Cerrahpaşa Faculty of Medicine, Istanbul University-Cerrahpaşa, 34098 Istanbul, Türkiye; drmuradguliyev@gmail.com (M.G.); drserkannebi@yahoo.com (N.S.D.); 18 Department of Medical Oncology, Faculty of Medicine, Tekirdağ Namık Kemal University, 59030 Tekirdağ, Türkiye; eyyupcavdar@hotmail.com (E.Ç.); omerfarukelcicek@gmail.com (Ö.F.E.); 19 Department of Medical Oncology, Mersin City Hospital, 33040 Mersin, Türkiye; dr.ainal@gmail.com; 20 Department of Medical Oncology, Faculty of Medicine, Ankara University, 06560 Ankara, Türkiye; pinar_kubilay@hotmail.com; 21 Department of Medical Oncology, Faculty of Medicine, Gaziantep University, 27310 Gaziantep, Türkiye; kalemali88@gmail.com (A.K.); drtulaykus83@hotmail.com (T.K.); 22 Department of Medical Oncology, Faculty of Medicine, Akdeniz University, 07070 Antalya, Türkiye; aysegul_merc@hotmail.com (A.M.Ç.); semasezgingoksu@gmail.com (S.S.G.); 23 Department of Medical Oncology, Koç University Hospital, 34010 Istanbul, Türkiye; sinem_kocak@yahoo.com (S.A.); fselcukbiricik@ku.edu.tr (F.S.); 24 Department of Medical Oncology, Faculty of Medicine, Cukurova University Balcalı Hospital, 01330 Adana, Türkiye; sedatb23@hotmail.com (S.B.); tkoseci@cu.edu.tr (T.K.); 25 Department of Medical Oncology, Faculty of Medicine, Muğla Sıtkı Koçman University, 48000 Muğla, Türkiye; kitaplisait@gmail.com (S.K.); dr.ozgur.tanriverdi@gmail.com (Ö.T.); 26 Department of Medical Oncology, Afyonkarahisar Health Sciences University, 03030 Afyonkarahisar, Türkiye; mervekuday@gmail.com; 27 Department of Medical Oncology, Bezmiâlem Foundation University, 34093 Istanbul, Türkiye; lamia.seker1@gmail.com; 28 Department of Medical Oncology, Vm Medikalpark Maltepe Hospital, 34846 Istanbul, Türkiye; nergiz.mecidova1991@gmail.com; 29 Department of Medical Oncology, Faculty of Medicine, Ege University, 35100 Izmir, Türkiye; hasan-cagri@windowslive.com; 30 Department of Medical Oncology, SBU Tepecik Training and Research Hospital, 35020 Izmir, Türkiye; memed.uzun3846@gmail.com; 31 Department of Medical Oncology, Göztepe City Hospital, Istanbul Medeniyet University, 34722 Istanbul, Türkiye; ilkernihat@gmail.com; 32 Department of Public Health, Faculty of Medicine, Çukurova University, 01330 Adana, Türkiye; bmete@cu.edu.tr

**Keywords:** gastric cancer, perioperative FLOT, post-FLOT recurrence, first-line systemic therapy, relapse timing, HER2-positive, real-world evidence

## Abstract

*Background and Objectives*: Perioperative fluorouracil, leucovorin, oxaliplatin, and docetaxel (FLOT) is the standard of care for resectable gastric and gastroesophageal junction adenocarcinoma; however, up to 50% of patients develop metastatic recurrence. These patients have prior exposure to platinum and taxane agents, and optimal first-line treatment in the metastatic setting remains undefined. This study aimed to characterize real-world treatment patterns and outcomes in patients progressing after perioperative FLOT, focusing on relapse timing and HER2 status. *Materials and Methods*: This retrospective, multicenter cohort study included 296 patients from 31 centers across Türkiye, stratified into early relapse (≤6 months, n = 114) and late relapse (>6 months, n = 182) groups. Survival analyses were performed using the Kaplan-Meier method and Cox proportional hazards regression. Primary endpoints were progression-free survival (PFS) and overall survival (OS). *Results*: Median PFS and OS for the entire cohort were 6 and 9 months, respectively. Early relapsers had significantly shorter median PFS (4 vs. 6 months, *p* = 0.029) and OS (8 vs. 12 months, *p* = 0.047); however, early relapse timing did not retain independent prognostic significance on multivariable analysis. No significant difference in PFS or OS was observed between cytotoxic chemotherapy regimens in either relapse group. HER2 positivity was the only independent predictor of improved PFS on multivariable Cox analysis (HR 0.48, 95% CI 0.29–0.81; *p* = 0.006). In the late relapse group, trastuzumab-based chemotherapy achieved a median PFS of 14 months and OS of 18 months, significantly superior to all cytotoxic regimens (PFS *p* = 0.007; OS *p* = 0.029). *Conclusions*: In patients progressing after perioperative FLOT, cytotoxic chemotherapy regimen selection did not demonstrate a statistically significant survival difference in this retrospective cohort, regardless of relapse timing. HER2 positivity is the dominant predictive biomarker, and trastuzumab-based therapy suggests a potential survival benefit that warrants prospective validation. Comprehensive biomarker profiling at metastatic diagnosis and prospective trials designed for this post-FLOT population are needed to establish evidence-based treatment standards.

## 1. Introduction

Gastric and gastroesophageal junction (GEJ) cancers remain among the most lethal malignancies worldwide, representing the fifth most diagnosed cancer and accounting for approximately 770,000 deaths annually [1]. The majority of patients are diagnosed at locally advanced or metastatic stages, and even those who undergo curative-intent surgery face a high risk of disease recurrence [2].

For patients with resectable locally advanced gastric and GEJ adenocarcinoma, perioperative chemotherapy (ChT) has become the cornerstone of treatment in current guidelines. The landmark FLOT4 trial established the superiority of perioperative docetaxel, oxaliplatin, leucovorin, and 5-fluorouracil (FLOT) over epirubicin-based regimens, demonstrating significantly improved pathological complete response rates, progression-free survival, and overall survival [3]. Real-world data from both European and Turkish cohorts have subsequently confirmed the feasibility and efficacy of perioperative FLOT in routine clinical practice [4,5,6]. A prior Turkish Oncology Group (TOG) study further characterized prognostic factors associated with perioperative FLOT outcomes in a real-world Turkish population, underscoring the relevance of pathological response and nodal status as key determinants of long-term outcomes [7]. More recently, the MATTERHORN trial demonstrated that the addition of durvalumab to perioperative FLOT significantly improved event-free survival in resectable gastric and GEJ adenocarcinoma, further expanding the perioperative treatment landscape [8]. As more patients receive multi-agent perioperative regimens incorporating platinum, taxane, and potentially immunotherapy agents, the population who subsequently relapses in the metastatic setting will continue to grow.

However, despite optimal perioperative treatment and curative resection, a substantial proportion of patients develop disease recurrence and ultimately progress to the metastatic setting. Real-world analyses have reported that recurrence occurs in up to 40–50% of patients following FLOT-based perioperative therapy, with peritoneal and hematogenous spread being the most common patterns [5,9]. This clinical scenario presents a unique and underappreciated challenge: patients who relapse after perioperative FLOT have already been exposed to platinum and taxane agents—the two most active drug classes in gastric cancer—leaving clinicians with limited evidence-based options for subsequent systemic therapy. Recent data suggest that outcomes following relapse are poor and more analogous to a second-line than a first-line treatment setting [10]. As recently emphasized in the first dedicated critical review of this topic, no randomized evidence currently exists to guide first-line treatment selection in patients previously exposed to perioperative FLOT, and treatment decisions in routine practice are largely extrapolated from trials that enrolled predominantly ChT-naive patients [11].

The landmark trials establishing current first-line metastatic treatment paradigms—including CheckMate 649, KEYNOTE-859, and KEYNOTE-811 [12,13,14]—enrolled patients defined as previously untreated in the metastatic setting and did not report outcomes separately for ChT-pretreated versus ChT-naive patients, leaving a critical evidence gap regarding the benefit of these regimens specifically in the post-FLOT setting.

An additional layer of complexity arises from the heterogeneity of relapse patterns following perioperative FLOT. Patients who experience disease recurrence within the first six months of completing treatment likely harbor inherently resistant tumors—a pattern analogous to platinum-refractory disease in ovarian cancer [15]—whereas those relapsing beyond six months may retain partial sensitivity to previously administered agents. This distinction in relapse timing carries potential prognostic and therapeutic implications; yet, to our knowledge, no study has systematically evaluated whether the timing of relapse after perioperative FLOT independently influences outcomes in the subsequent metastatic setting.

Beyond relapse timing, the molecular landscape of the tumor is increasingly recognized as a key determinant of treatment selection and prognosis. HER2-positive tumors account for approximately 10–15% of gastric cancers, and trastuzumab-based therapy has demonstrated a meaningful survival benefit in the first-line metastatic setting [1,2]. Similarly, microsatellite instability-high (MSI-H) status has emerged as a favorable prognostic marker and a predictor of response to immune checkpoint inhibitors, with the PROSECCO study demonstrating significantly improved disease-free and overall survival in MSI-H patients treated with perioperative FLOT [9]. Whether these biomarkers retain their prognostic relevance specifically in patients who progress after perioperative FLOT remains an open and clinically important question [16,17].

In this context, we conducted a multicenter, retrospective cohort study within the Turkish Oncology Group (TOG) to characterize real-world first-line treatment patterns and outcomes in patients with gastric or GEJ cancer who experienced disease progression following perioperative FLOT and subsequent curative resection. We further examined whether the timing of relapse—early (≤6 months) versus late (>6 months) after FLOT completion—serves as a prognostic determinant of first-line metastatic treatment outcomes. To our knowledge, this represents the one of largest multicenter real-world cohort specifically designed to address this clinically important and evidence-poor setting.

## 2. Materials and Methods

### 2.1. Study Design, Patient Selection and Ethics

This retrospective, multicenter cohort study included patients with histologically confirmed gastric or GEJ adenocarcinoma who experienced disease progression following perioperative FLOT ChT and underwent first-line systemic treatment in the metastatic setting between January 2018 and December 2024 at 31 participating centers across Türkiye. This study received ethical approval from the Kocaeli University Ethics Committee with the number GOKAEK-2024/04.18 (Project code: 2024/121) and was approved by the institutional review boards of all participating centers. The study was conducted in accordance with the principles of the Declaration of Helsinki. Due to the retrospective nature of the study, informed consent was waived.

Inclusion criteria comprised: (1) histologically confirmed gastric or GEJ adenocarcinoma; (2) four cycles of FLOT ChT followed by curative-intent surgical resection followed by four cycles FLOT ChT; (3) documented radiological or pathological evidence of metastatic recurrence following perioperative treatment; (4) initiation of at least one cycle of first-line systemic therapy in the metastatic setting; (5) age ≥18 years at the time of first-line metastatic treatment initiation; and (6) adequate baseline clinical, pathological, and radiological data available for outcome assessment.

Exclusion criteria included: (1) concurrent or prior malignancy other than gastric or GEJ adenocarcinoma; (2) insufficient follow-up data (fewer than one radiological or clinical assessment after treatment initiation); and (3) upfront metastatic disease without prior perioperative FLOT.

As this study captures real-world practice in the absence of standardized treatment protocols for the post-FLOT metastatic setting, treatment allocation was non-randomized and heterogeneous across centers, which is acknowledged as an inherent limitation of the study design.

### 2.2. Data Collection

Patient data were retrospectively extracted from institutional medical records at each participating center by the treating oncologist or a designated research team member. A standardized data collection form was used across all centers to ensure consistency in the variables recorded. Collected data were subsequently compiled and centrally reviewed by the coordinating center for completeness and internal consistency; implausible or inconsistent entries were flagged and resolved through direct communication with the submitting center. Collected variables included demographic and clinicopathological characteristics, perioperative treatment details, pathological response (ypT, ypN stage, pCR defined as ypT0N0), HER2 status, MSI status, ECOG performance status at initiation of first-line metastatic therapy, metastatic site(s) at relapse, first-line treatment regimen, best overall response, and survival outcomes.

### 2.3. Relapse Timing and Treatment Groups

Relapse timing was defined as the interval between completion of the final cycle of perioperative FLOT and radiological or clinical confirmation of disease recurrence. Based on this interval, patients were stratified into two groups: early relapse (≤6 months after FLOT completion) and late relapse (>6 months after FLOT completion). This threshold was selected based on its clinical relevance as a surrogate marker of platinum sensitivity and is consistent with definitions used in analogous contexts in other gastrointestinal malignancies.

First-line metastatic treatment regimens were classified into three subgroups within each relapse timing cohort. Grouping was based on the most frequently administered regimens in each cohort, as observed in routine clinical practice across participating centers, rather than protocol-driven assignment. In the early relapse group—where prior platinum exposure renders re-challenge less clinically appropriate—(1) FOLFIRI emerged as the predominant regimen (n = 52, 45.6%) and was therefore selected as the reference cytotoxic group, alongside (2) ChT+trastuzumab and (3) all other regimens. In the late relapse group—where partial platinum sensitivity may be retained—(1) FOLFOX/CAPEOX was the most administered regimen (n = 81, 44.5%) and served as the reference cytotoxic group, alongside (2) ChT+trastuzumab and (3) all other regimens. Treatment selection across centers was at the discretion of the treating oncologist, reflecting real-world clinical practice in the absence of established guidelines for this patient population.

### 2.4. Endpoints

The primary endpoints were progression-free survival (PFS) and overall survival (OS). PFS was defined as the time from initiation of first-line metastatic treatment to radiological or clinical disease progression or death from any cause, whichever occurred first. OS was defined as the time from initiation of first-line metastatic treatment to death from any cause. Patients alive and without disease progression at the time of last follow-up were censored. Tumor response was assessed by the treating oncologist at each center according to Response Evaluation Criteria in Solid Tumors (RECIST) version 1.1 based on cross-sectional imaging. Central radiology review was not performed. Secondary endpoints included objective response rate (ORR; proportion of patients achieving complete or partial response), disease control rate (DCR; proportion of patients achieving complete response, partial response, or stable disease), and identification of independent prognostic factors for survival.

### 2.5. Statistical Analysis

Continuous variables were summarized as mean ± standard deviation (SD) or median (interquartile range) as appropriate; categorical variables are presented as frequencies and percentages. Normality was assessed using the Shapiro-Wilk test. Between-group comparisons were performed using the independent samples *t*-test or Mann-Whitney U test for continuous variables, and the chi-square or Fisher’s exact test for categorical variables, as appropriate. Survival probabilities were estimated using the Kaplan-Meier method and between-group differences assessed using the log-rank test. For comparisons involving three treatment subgroups, the multivariate log-rank test was applied for overall comparison, followed by pairwise log-rank tests for post-hoc analysis. Median survival times and 95% confidence intervals (CI) are reported throughout. Univariable and multivariable Cox proportional hazards regression analyses were performed to identify independent prognostic factors for PFS and OS. Variables with *p* < 0.10 on univariable analysis were eligible for inclusion in the multivariable model. Formal assessment of collinearity between covariates was not performed. Results are expressed as hazard ratios (HR) with 95% CI. A two-sided *p*-value of <0.05 was considered statistically significant. Missing data were not imputed; variables with substantial missing rates, including MSI status and PD-L1 expression, were excluded from multivariable models. No formal sensitivity analyses were performed. The primary analyses—PFS and OS stratified by relapse timing—were pre-specified. All subgroup analyses, including those involving HER2 status, the three-group survival comparison, and treatment-specific comparisons within each relapse timing cohort, were exploratory in nature. No correction for multiple comparisons was applied; accordingly, these findings should be interpreted with caution as hypothesis-generating. Statistical analyses were performed using the Jamovi software (The Jamovi Project, Version 2.6.44).

## 3. Results

A total of 296 patients from 31 centers were included; 114 (38.5%) experienced early relapse (≤6 months) and 182 (61.5%) late relapse (>6 months). Baseline characteristics are presented in Table 1. Patients in the early relapse group were significantly younger (mean 57.1 vs. 60.2 years, *p* = 0.012) and had a higher proportion of poorly differentiated tumors (70.2% vs. 46.7%, *p* < 0.001). Pathological staging after surgery revealed a markedly higher rate of ypTNM stage III disease in the early relapse group (82.5% vs. 61.5%, *p* < 0.001), while pathological complete or near-complete responses were more frequent in the late relapse group (14.3% vs. 4.4%). Sex, comorbidity, ECOG performance status, HER2 status, MSI status, tumor localization, and metastatic site distribution were comparable between the two groups. Peritoneal metastasis was the most common site of distant disease, observed in 57.8% of all patients.

First-line metastatic treatment regimens are summarized in Table 2. FOLFIRI predominated in the early relapse group (45.6%) and FOLFOX/CAPEOX in the late relapse group (44.5%); ChT+Trastuzumab was administered exclusively to HER2-positive patients in both groups.

**Figure 1:**
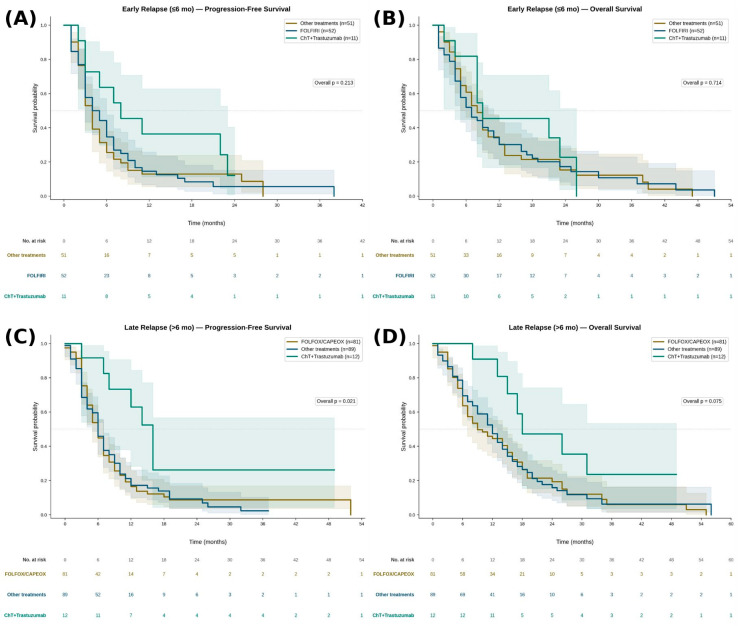
Kaplan-Meier survival curves for first-line metastatic treatment groups stratified by relapse timing after perioperative FLOT. (**A**) PFS in the early relapse group (≤6 months); (**B**) OS in the early relapse group; (**C**) PFS in the late relapse group (>6 months); (**D**) OS in the late relapse group. Shaded areas represent 95% confidence intervals. Numbers at risk are displayed below each panel. Overall log-rank *p*-values are shown in each panel; pairwise comparisons were performed between ChT+Trastuzumab and each cytotoxic group. FOLFIRI: fluorouracil + leucovorin + irinotecan; FOLFOX: fluorouracil + leucovorin + oxaliplatin; CAPEOX: capecitabine + oxaliplatin; ChT: chemotherapy.

Over a median follow-up of 16 months (maximum 56 months), the median PFS and OS for the entire cohort were 6 months and 9 months, respectively. Patients with early relapse demonstrated significantly shorter PFS (4 vs. 6 months, *p* = 0.029) and OS (8 vs. 12 months, *p* = 0.047) compared to those with late relapse. First-line treatment regimens and survival outcomes stratified by relapse timing are presented in Figure 1A–D. In patients with early relapse, median PFS was 4 months in both the FOLFIRI and other treatments groups, and 8 months in the ChT+Trastuzumab group (overall *p* = 0.213). Median OS was 7, 8, and 9 months, respectively (overall *p* = 0.714). No statistically significant difference in PFS or OS was observed between FOLFIRI and other treatments (*p* = 0.672 and *p* = 0.902, respectively). Although the ChT+Trastuzumab group demonstrated numerically longer PFS and OS, this did not reach statistical significance, likely reflecting the limited sample size of this subgroup. In patients with late relapse, median PFS was 6 months in both the FOLFOX/CAPEOX and other treatments groups, and 14 months in the ChT+Trastuzumab group (overall *p* = 0.021). Median OS was 9, 12, and 18 months, respectively (overall *p* = 0.075). Pairwise analysis demonstrated significantly longer PFS with ChT+Trastuzumab compared to both FOLFOX/CAPEOX (*p* = 0.007) and other treatments (*p* = 0.013). OS was also significantly longer with ChT+Trastuzumab versus FOLFOX/CAPEOX (*p* = 0.029). No significant difference in PFS or OS was observed between FOLFOX/CAPEOX and other treatments (*p* = 0.986 and *p* = 0.827, respectively).

**Figure 2:**
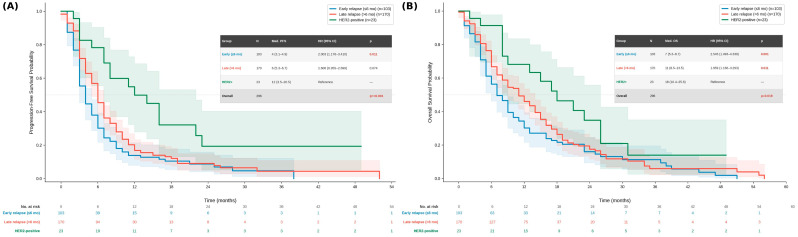
Survival analysis by relapse timing and HER2 status after perioperative FLOT (n = 296). (**A**) PFS and (**B**) OS comparing HER2-negative early relapsers (n = 103), HER2-negative late relapsers (n = 170), and HER2-positive patients (n = 23). Embedded tables display median survival, HR with 95% CI, and *p*-values, with HER2-positive patients as the reference group. Shaded areas represent 95% confidence intervals. Numbers at risk are displayed below each panel. HER2: human epidermal growth factor receptor 2; PFS: Progression-free survival; OS: overall survival; CI: confidence interval; HR: hazard ratio.

To further characterize the prognostic impact of HER2 status independent of relapse timing, a three-group survival analysis was performed comparing HER2-negative early relapsers, HER2-negative late relapsers, and all HER2-positive patients (Figure 2A,B). HER2-positive patients demonstrated markedly superior outcomes compared to both HER2-negative groups. Median PFS was 12 months (95% CI 3.5–20.5) in the HER2-positive group, compared to 6 months (95% CI 5.3–6.7) in late relapsers and 4 months (95% CI 3.1–4.9) in early relapsers (overall log-rank *p* < 0.001). On Cox regression, early relapse was associated with a significantly higher risk of progression compared to HER2-positive patients (HR 2.003, 95% CI 1.176–3.410; *p* = 0.011), as was late relapse (HR 1.606, 95% CI 0.955–2.699; *p* = 0.074). Similarly, median OS was 18 months (95% CI 10.4–25.6) in HER2-positive patients, compared to 11 months (95% CI 8.5–13.5) in late relapsers and 7 months (95% CI 5.3–8.7) in early relapsers (overall log-rank *p* = 0.018). Both early relapse (HR 2.545, 95% CI 1.493–4.339; *p* = 0.001) and late relapse (HR 1.959, 95% CI 1.166–3.293; *p* = 0.011) were independently associated with significantly worse OS compared to HER2-positive patients. These findings demonstrate that HER2 positivity confers a survival advantage independent of relapse timing.

**Figure 3:**
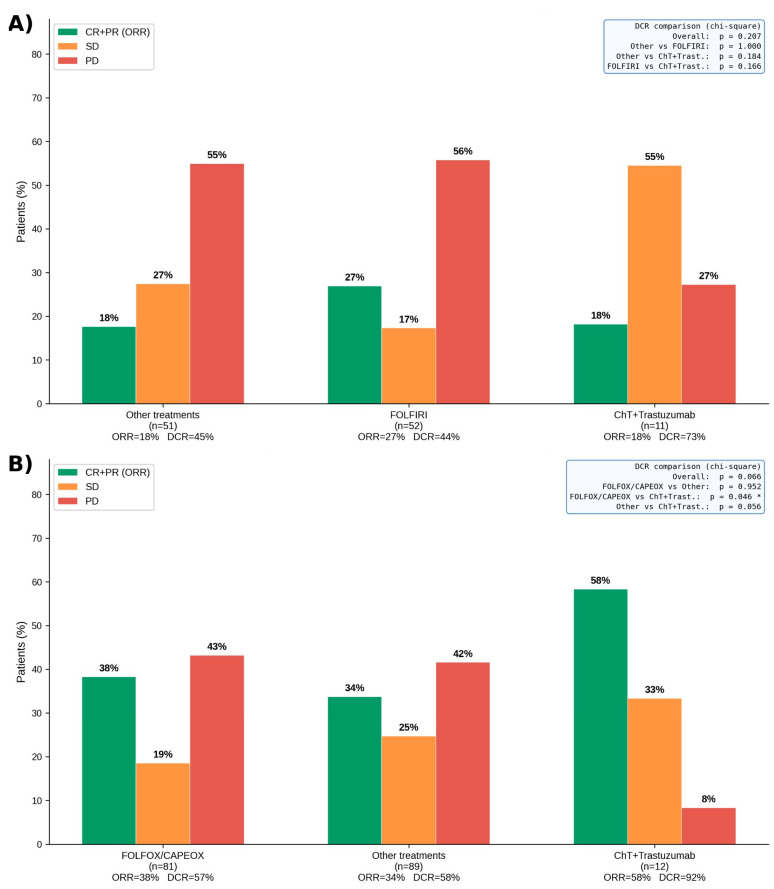
ORR and DCR stratified by treatment regimen and relapse timing after perioperative FLOT. (**A**) Early relapse group (≤6 months); (**B**) Late relapse group (>6 months). Pairwise DCR comparisons were performed using the chi-square test; *p*-values are displayed in the inset boxes. ORR and DCR values are shown below each treatment group. ORR: objective response rate (CR + PR); DCR: disease control rate (CR + PR + SD); CR: complete response; PR: partial response; SD: stable disease; PD: progressive disease; FOLFIRI: fluorouracil + leucovorin + irinotecan; FOLFOX: fluorouracil + leucovorin + oxaliplatin; CAPEOX: capecitabine + oxaliplatin; ChT: chemotherapy. * *p* < 0.05.

Objective response rates (ORR) and disease control rates (DCR) stratified by relapse timing and treatment regimen are illustrated in Figure 3A,B. In the early relapse group, no statistically significant differences in ORR or DCR were observed across the three treatment groups (overall DCR *p* = 0.207). In the late relapse group, ChT+Trastuzumab achieved a significantly higher DCR compared to FOLFOX/CAPEOX (92% vs. 57%, *p* = 0.046), while no significant difference was observed between cytotoxic regimens (overall DCR *p* = 0.066).

Univariable and multivariable Cox proportional hazards regression analyses for PFS are presented in Table 3. On univariable analysis, early relapse (HR 1.32, 95% CI 1.03–1.69; *p* = 0.029), liver metastasis (HR 1.35, 95% CI 1.04–1.76; *p* = 0.026), and HER2 positivity (HR 0.45, 95% CI 0.27–0.74; *p* = 0.002) were significantly associated with PFS. Grade 3 histology (*p* = 0.078), ECOG score ≥ 2 (*p* = 0.057), and signet ring cell histology (*p* = 0.065) showed borderline associations. On multivariable analysis, HER2 positivity remained the only independent predictor of improved PFS (HR 0.48, 95% CI 0.29–0.81; *p* = 0.006), reflecting the benefit of trastuzumab-based therapy in this subgroup. Early relapse timing did not reach statistical significance as an independent prognostic factor after adjustment (HR 1.26, 95% CI 0.97–1.63; *p* = 0.086), suggesting that its prognostic effect may be partially mediated by other tumor characteristics such as grade and histological subtype.

Univariable and multivariable Cox regression analyses for OS are presented in Table 4. On univariable analysis, early relapse (HR 1.29, 95% CI 1.00–1.67; *p* = 0.047), grade 3 histology (HR 1.43, 95% CI 1.11–1.85; *p* = 0.006), HER2 positivity (HR 0.57, 95% CI 0.34–0.94; *p* = 0.028), and signet ring cell histology (HR 1.50, 95% CI 1.07–2.10; *p* = 0.018) were significantly associated with OS. On multivariable analysis, grade 3 histology (HR 1.32, 95% CI 1.01–1.72; *p* = 0.045) emerged as an independent adverse prognostic factor for OS. HER2 positivity showed a trend toward improved OS on multivariable analysis (HR 0.68, 95% CI 0.40–1.13; *p* = 0.138), though this did not retain statistical significance, likely due to the limited number of HER2-positive patients (n = 23). Signet ring cell histology similarly showed a borderline association (HR 1.36, 95% CI 0.97–1.92; *p* = 0.078). Early relapse timing did not independently predict OS after multivariable adjustment (HR 1.14, 95% CI 0.87–1.49; *p* = 0.335). Taken together, the Cox analyses indicate that among patients progressing after perioperative FLOT, tumor grade and HER2 status are the key determinants of survival outcomes, while relapse timing exerts a prognostic effect primarily through its correlation with aggressive tumor biology rather than as an independent predictor.

## 4. Discussion

This multicenter, retrospective cohort study of 296 patients with gastric or GEJ adenocarcinoma progressing after perioperative FLOT reports three principal findings. First, outcomes in this post-FLOT metastatic population are uniformly poor—with a median PFS of 6 months and OS of 9 months for the entire cohort—and no statistically significant survival difference was observed between cytotoxic ChT regimens in either relapse timing group. Second, while early relapse was associated with significantly shorter median PFS (4 vs. 6 months, *p* = 0.029) and OS (8 vs. 12 months, *p* = 0.047) compared to late relapse on Kaplan-Meier analysis, early relapse timing did not retain independent prognostic significance on multivariable Cox regression for either PFS or OS after adjustment for tumor grade, HER2 status, and histological subtype, suggesting that its apparent prognostic value is largely driven by underlying tumor biology rather than relapse timing per se. Third, HER2-positive patients treated with trastuzumab-containing ChT demonstrated markedly superior outcomes compared to HER2-negative patients in both relapse timing groups, and HER2 positivity was the only independent predictor of improved PFS on multivariable analysis (HR 0.48, 95% CI 0.29–0.81; *p* = 0.006), a benefit that transcended the prognostic impact of relapse timing. Importantly, this study addresses a clinically underdefined population for which no dedicated guideline recommendations currently exist, highlighting a critical gap between randomized trial evidence and real-world clinical decision-making.

The clinical scenario addressed in this study is fundamentally distinct from the treatment-naïve metastatic setting that has been the focus of landmark randomized trials. Patients who relapse after perioperative FLOT have already been exposed to platinum, taxane, and fluoropyrimidine agents, the most active cytotoxic classes in gastric cancer, leaving clinicians with very limited evidence-based guidance for subsequent therapy [1,2]. In our cohort, peritoneal metastasis was present in 57.8% of all patients at the time of metastatic diagnosis, a rate substantially higher than that observed in treatment-naïve metastatic populations and reflective of the aggressive biology of post-FLOT relapse. Epithelial-mesenchymal transition has been proposed as a key biological mechanism underlying this aggressive phenotype, driving both peritoneal dissemination and intrinsic chemotherapy resistance in gastric cancer [18]. A national Danish cohort study of 89 patients relapsing after perioperative ChT and radical resection reported a median OS of only 4.6 months from the date of recurrence and concluded that the efficacy of palliative ChT at relapse was most comparable to that of second-line rather than first-line treatment in ChT-naive patients [10]. Our findings, with a median OS of 8 and 12 months in early and late relapsers, respectively, align with and extend these observations. This further supports that post-FLOT relapse represents a distinct pretreated metastatic disease state.

The failure of early relapse timing to independently predict survival after multivariable adjustment is biologically coherent and clinically important. Early relapsers in our cohort had a significantly higher prevalence of poorly differentiated tumors (70.2% vs. 46.7%, *p* < 0.001) and ypTNM stage III disease (82.5% vs. 61.5%, *p* < 0.001) compared to late relapsers, findings consistent with those of Dayanamby et al., who identified ypN3 stage as the strongest independent predictor of early recurrence after neoadjuvant FLOT in a dedicated retrospective analysis [19]. Poor pathological response predicts worse outcomes. Taken together, these data indicate that early relapse after FLOT is primarily a marker of aggressive tumor phenotype rather than an independent biological entity, and that relapse timing alone should not drive treatment selection in the first-line metastatic setting.

The equivalence of cytotoxic ChT regimens across both relapse timing cohorts is a central finding of our study. In the early relapse group, FOLFIRI was the most commonly administered regimen (n = 52, 45.6%), while FOLFOX/CAPEOX predominated in the late relapse group (n = 81, 44.5%), reflecting clinician-driven assumptions about platinum sensitivity. Despite these differences in treatment selection, no statistically significant differences in PFS or OS were observed between cytotoxic regimens in either cohort (early relapse: PFS *p* = 0.672, OS *p* = 0.902; late relapse: PFS *p* = 0.986, OS *p* = 0.827). These findings are corroborated by the largest multicenter retrospective study addressing ChT after early relapse following adjuvant S-1 monotherapy in Japan, which enrolled 207 patients and found no statistically significant difference in OS between fluoropyrimidine-platinum, taxane-based, and irinotecan-based regimens on multivariable analysis [20]. The original observation by Shitara et al. that S-1 plus cisplatin was largely ineffective in patients relapsing within six months of adjuvant S-1—with a response rate of only 5% and median OS of 7.3 months compared to 37.5% and 16.6 months in late relapsers—established the foundational concept that early post-adjuvant relapse confers inherent ChT resistance that transcends regimen selection [21]. Mitani et al. subsequently confirmed poor outcomes across all ChT classes in a multicenter retrospective cohort of early relapsers after adjuvant S-1, with a median OS of 11.4 months regardless of the regimen administered [22]. Although perioperative FLOT involves substantially greater prior drug exposure than adjuvant S-1 monotherapy, the biological conclusion is consistent across these datasets: in patients relapsing after prior systemic therapy for gastric cancer, the choice of cytotoxic backbone does not meaningfully alter survival. These findings suggest that patients progressing after perioperative FLOT biologically resemble a second-line population rather than treatment-naïve metastatic disease, which has important implications for treatment selection and trial design.

Regarding the role of irinotecan-based regimens specifically in the post-FLOT setting, the phase II RAMIRIS trial demonstrated that FOLFIRI plus ramucirumab achieved a higher ORR (25% vs. 8%) and a longer median PFS (4.6 vs. 2.1 months) compared to paclitaxel plus ramucirumab in docetaxel-pretreated patients [23]. These findings provided biological justification for preferring irinotecan-based therapy after FLOT exposure, given the absence of prior irinotecan use and concerns about taxane cross-resistance. However, in our cohort, FOLFIRI as a single-agent irinotecan-containing backbone without ramucirumab did not demonstrate superior outcomes compared to other regimens in the early relapse group. Whether the addition of ramucirumab to irinotecan-based ChT would improve outcomes specifically in the post-FLOT first-line setting remains an important unanswered question that warrants prospective investigation. The XParTS-I phase II trial, which evaluated capecitabine plus cisplatin in patients with early relapse after adjuvant S-1, reported a median PFS of 4.4 months and OS of 13.7 months, outcomes substantially inferior to those observed in treatment-naïve first-line studies, and further underscoring the ChT-resistant phenotype of early-relapsing gastric cancer [24].

The most clinically actionable finding of our study is the differential impact of HER2-directed therapy. In the late relapse group, HER2-positive patients receiving ChT+Trastuzumab (n = 12) achieved a median PFS of 14 months and OS of 18 months, significantly superior to both FOLFOX/CAPEOX (PFS *p* = 0.007, OS *p* = 0.029) and other treatments (PFS *p* = 0.013), with a DCR of 91.7% versus 57–59% in cytotoxic groups (*p* = 0.046 vs. FOLFOX/CAPEOX). In the early relapse group, ChT+Trastuzumab (n = 11) demonstrated numerically superior PFS (8 months), OS (9 months), and DCR (73% vs. 44–45%), though differences did not reach statistical significance, likely reflecting the limited sample size. In the three-group analysis, HER2-positive patients (n = 23) demonstrated markedly superior median PFS (12 months) and OS (18 months) compared to HER2-negative late relapsers (PFS 6 months, OS 11 months) and HER2-negative early relapsers (PFS 4 months, OS 7 months), with both relapse groups showing significantly worse outcomes versus HER2-positive patients on Cox regression (early relapse PFS HR 2.003, *p* = 0.011; OS HR 2.545, *p* = 0.001; late relapse OS HR 1.959, *p* = 0.011). These findings identify HER2 as a potentially important predictive biomarker in the post-FLOT metastatic setting; however, given the small subgroup size (n = 23), these findings should be considered hypothesis-generating and require prospective validation.

The survival benefit of trastuzumab in HER2-positive advanced gastric cancer was established by the ToGA trial, which demonstrated a significant OS advantage with trastuzumab plus ChT versus ChT alone (13.8 vs. 11.1 months; HR 0.74; *p* = 0.0046) in a ChT-naive population [25]. Our data extend this observation to the post-FLOT setting, where trastuzumab-based therapy appears to retain clinically meaningful efficacy despite prior multiagent ChT exposure. Notably, HER2 expression in gastric cancer is characterized by significant intratumoral and intertumoral heterogeneity, with discordance between primary and metastatic sites reported in up to 30% of cases [26], and HER2 status can further evolve between primary resection and metastatic relapse due to clonal evolution or treatment-induced selection pressure [27]. These considerations underscore the critical importance of systematic HER2 re-testing at the time of metastatic diagnosis in all post-FLOT patients, regardless of the HER2 status recorded at initial surgery. Modern HER2-directed combinations such as pembrolizumab plus trastuzumab and ChT, as established in KEYNOTE-811 [14] have not been evaluated in the post-perioperative-FLOT setting, and whether the incremental benefit of adding PD-1 inhibition to trastuzumab-based therapy persists after prior FLOT exposure remains entirely unknown.

The role of MSI-H status and immune checkpoint inhibitors in this population warrants specific consideration. In our cohort, only 12 patients (4.1%) were identified as MSI-H, and the majority did not receive immune checkpoint inhibitor-containing regimens in the first-line metastatic setting. The PROSECCO study demonstrated that MSI-H status is associated with significantly improved disease-free and overall survival in patients treated with perioperative FLOT, suggesting that this subgroup retains a favorable tumor biology even in the context of prior ChT exposure [9]. The five-year follow-up of CheckMate 649 confirmed that patients with MSI-H tumors derive the greatest magnitude of OS benefit from PD-1 inhibitor plus ChT, with a hazard ratio of 0.37 (95% CI 0.18–0.75) compared to microsatellite-stable patients [28]. Whether MSI-H patients who do relapse after perioperative FLOT retain robust sensitivity to checkpoint inhibition in the first-line metastatic setting remains an unanswered and important clinical question. The underutilization of immunotherapy in MSI-H patients in our cohort likely reflects the study period and regional access limitations and represents a correctable gap in clinical practice. Given the incomplete MSI data in the present cohort, firm conclusions regarding the role of MSI-H status in post-FLOT metastatic disease cannot be drawn from this study. Nevertheless, universal MSI testing at metastatic diagnosis should be considered a priority in future prospective studies.

On multivariable Cox analysis for OS, grade 3 poorly differentiated histology was the only independent adverse prognostic factor (HR 1.32, 95% CI 1.01–1.72; *p* = 0.045). The independent prognostic significance of tumor grade is consistent with the well-established poor prognosis of poorly differentiated gastric cancer and its higher propensity for aggressive recurrence patterns, including peritoneal carcinomatosis [5]. Signet ring cell histology showed a borderline adverse association with OS on both univariable (HR 1.50, 95% CI 1.07–2.10; *p* = 0.018) and multivariable analyses (HR 1.36, 95% CI 0.97–1.92; *p* = 0.078), consistent with its established poor prognosis in gastric cancer and its higher prevalence in the early relapse group (18.4% vs. 12.1%) in our cohort.

Several limitations of this study must be acknowledged. The retrospective design introduces the potential for selection bias and incomplete data capture. Biomarker ascertainment was notably incomplete: MSI status was unknown in 71 patients (24.0%), and PD-L1 expression data were not systematically collected across centers. This limits the ability to draw meaningful conclusions regarding the role of these biomarkers in the post-FLOT setting, and the biomarker-driven interpretations presented in this manuscript should be considered in this context. The HER2-positive and MSI-H subgroups were small (n = 23 and n = 12, respectively), limiting the statistical power of subgroup analyses and precluding definitive conclusions. The heterogeneity of the other treatment category, which encompasses taxane-based combinations, DCF, checkpoint inhibitor-containing regimens, and ramucirumab-based combinations, limits granular interpretation of outcomes within this group. Furthermore, although the multivariable Cox regression models included all available clinically relevant confounders meeting the prespecified threshold, non-randomized physician-driven treatment allocation introduces the potential for residual unmeasured confounding that cannot be fully eliminated through statistical adjustment. No formal sensitivity analyses were performed to test the robustness of the primary findings. Additionally, collinearity between covariates in the multivariable models was not formally assessed, and central radiology review for response evaluation was not performed, representing further inherent limitations of the multicenter retrospective design. Finally, data on subsequent lines of therapy were not systematically collected, precluding assessment of the impact of treatment sequencing on overall outcomes.

## 5. Conclusions

Patients with gastric or GEJ adenocarcinoma progressing after perioperative FLOT represent a clinically challenging population with poor outcomes in the first-line metastatic setting. Cytotoxic chemotherapy backbone selection did not demonstrate a significant survival difference in this retrospective analysis, regardless of relapse timing, while early relapse primarily reflects aggressive tumor biology rather than an independent prognostic factor. In contrast, HER2-positive patients appeared to derive a survival benefit from trastuzumab-based therapy in this hypothesis-generating analysis, highlighting HER2 as a potentially relevant predictive biomarker that warrants further prospective investigation. These findings support routine HER2 re-testing and comprehensive biomarker profiling at relapse, and underscore the need for prospective studies with systematic and complete biomarker profiling to establish evidence-based, biomarker-driven treatment strategies in the post-FLOT population.

## Figures and Tables

**Table 1:** Baseline characteristics of patients stratified by relapse timing after perioperative FLOT.

Variable		All Patients (n = 296)	≤6 Months (n = 114)	>6 Months (n = 182)	*p*
**Age** (Mean)		59.0 (10.4)	57.1 (11.2)	60.2 (9.7)	**0.012**
**Sex**	Female	83 (28.0)	33 (28.9)	50 (27.5)	0.887
	Male	213 (72.0)	81 (71.1)	132 (72.5)	
**Comorbidities**	Absent	204 (68.9)	79 (69.3)	125 (68.7)	1.000
	Present	92 (31.1)	35 (30.7)	57 (31.3)	
**Stage at diagnosis**	I	1 (0.3)	0 (0.0)	1 (0.5)	0.059
	IIA	22 (7.4)	7 (6.1)	15 (8.2)	
	IIB	38 (12.8)	8 (7.0)	30 (16.5)	
	III	231 (78.0)	96 (84.2)	135 (74.2)	
	IVA	4 (1.4)	3 (2.6)	1 (0.5)	
**Tumor histology**	Adenocarcinoma	239 (80.7)	92 (80.7)	147 (80.8)	**0.021**
	Signet-ring cell	43 (14.5)	21 (18.4)	22 (12.1)	
	Mucinous	14 (4.7)	1 (0.9)	13 (7.1)	
**Grade**	I	22 (7.4)	5 (4.4)	17 (9.3)	**<0.001**
	II	109 (36.8)	29 (25.4)	80 (44.0)	
	III	165 (55.7)	80 (70.2)	85 (46.7)	
**Tumor location**	GEJ	28 (9.5)	10 (8.8)	18 (9.9)	0.326
	Cardia	80 (27.0)	27 (23.7)	53 (29.1)	
	Fundus	19 (6.4)	10 (8.8)	9 (4.9)	
	Body	80 (27.0)	36 (31.6)	44 (24.2)	
	Antrum	84 (28.4)	28 (24.6)	56 (30.8)	
	Pylorus	5 (1.7)	3 (2.6)	2 (1.1)	
**ypTNM**	0	9 (3.0)	1 (0.9)	8 (4.4)	**<0.001**
	I	22 (7.4)	4 (3.5)	18 (9.9)	
	II	57 (19.3)	13 (11.4)	44 (24.2)	
	III	206 (69.6)	94 (82.5)	112 (61.5)	
**HER2 status**	Negative	273 (92.2)	103 (90.4)	170 (93.4)	0.464
	Positive	23 (7.8)	11 (9.6)	12 (6.6)	
**MSI status**	Stable	213 (72.0)	90 (78.9)	123 (67.6)	0.101
	High	12 (4.1)	3 (2.6)	9 (4.9)	
	Unknown	71 (24.0)	21 (18.4)	50 (27.5)	
**ECOG score**	0	136 (45.9)	49 (43.0)	87 (47.8)	0.371
	1	139 (47.0)	54 (47.4)	85 (46.7)	
	2	17 (5.7)	8 (7.0)	9 (4.9)	
	3	4 (1.4)	3 (2.6)	1 (0.5)	
**Liver Metastasis**		86 (29.1)	38 (33.3)	48 (26.4)	0.249
**Lung Metastasis**		54 (18.2)	19 (16.7)	35 (19.2)	0.688
**Peritoneum Metastasis**		171 (57.8)	67 (58.8)	104 (57.1)	0.877
**Bone Metastasis**		37 (12.5)	9 (7.9)	28 (15.4)	0.086
**Brain Metastasis**		3 (1.0)	2 (1.8)	1 (0.5)	0.681

GEJ: gastroesophageal junction; ypTNM: postoperative pathological tumor-node-metastasis stage; HER2: human epidermal growth factor receptor 2; MSI: microsatellite instability; ECOG: Eastern Cooperative Oncology Group. Data are presented as mean (standard deviation) for continuous variables and n (%) for categorical variables. Bold *p*-values indicate statistical significance (*p* < 0.05).

**Table 2:** First-line treatment regimens stratified by relapse timing after perioperative FLOT.

Early Relapse (≤6 Months) n = 114			Late Relapse (>6 Months) n = 182		
Treatment Regimen	n	%	Treatment Regimen	n	%
FOLFIRI	52	45.6%	FOLFOX/CAPEOX	81	44.5%
FOLFOX/CAPEOX	12	10.5%	FOLFIRI	39	21.4%
Paclitaxel	10	8.8%	DCF	9	4.9%
Other ChT regimen	10	8.8%	Paclitaxel + Carboplatin	9	4.9%
DCF	7	6.1%	IO/ChT+IO	7	3.8%
Paclitaxel + Carboplatin	6	5.3%	Paclitaxel	8	4.4%
IO/ChT+IO	5	4.4%	ChT + Ramucirumab	6	3.3%
ChT + Ramucirumab	1	0.9%	Other ChT regimen	11	6.0%
ChT + Trastuzumab	11	9.6%	ChT + Trastuzumab	12	6.6%
**Total**	**114**	**100%**	**Total**	**182**	**100%**

FOLFIRI: fluorouracil + leucovorin + irinotecan; FOLFOX: fluorouracil + leucovorin + oxaliplatin; CAPEOX: capecitabine + oxaliplatin; DCF: docetaxel + cisplatin + 5-fluorouracil; IO: immune checkpoint inhibitor; ChT: chemotherapy; HER2: human epidermal growth factor receptor 2. ChT+Trastuzumab was administered exclusively to HER2-positive patients.

**Table 3:** Univariable and multivariable Cox regression analysis for progression-free survival.

Variable	Univariable HR (95% CI)	*p*	Multivariable HR (95% CI)	*p*
Early relapse (≤6 mo)	1.32 (1.03–1.69)	**0.029**	1.26 (0.97–1.63)	0.086
Age ≥ 65 years	0.94 (0.72–1.23)	0.648	—	—
Male sex	0.97 (0.74–1.28)	0.849	—	—
ECOG score ≥ 2	1.56 (0.99–2.46)	0.057	—	—
Grade 3 (poor diff.)	1.25 (0.98–1.60)	0.078	1.06 (0.81–1.37)	0.675
ypTNM stage III	1.20 (0.92–1.56)	0.189	—	—
Peritoneal metastasis	1.10 (0.86–1.41)	0.456	—	—
Liver metastasis	1.35 (1.04–1.76)	**0.026**	—	—
HER2 positive	0.45 (0.27–0.74)	**0.002**	0.48 (0.29–0.81)	**0.006**
Signet ring cell histology	1.37 (0.98–1.91)	0.065	1.29 (0.92–1.82)	0.144

Multivariable model includes variables with *p* < 0.10 on univariable analysis: early relapse, ECOG PS ≥ 2, grade 3, HER2 positive, and signet ring cell histology. Bold values indicate statistical significance (*p* < 0.05). HR: hazard ratio; CI: confidence interval; ECOG: Eastern Cooperative Oncology Group performance status; ypTNM: postoperative pathological tumor-node-metastasis stage; HER2: human epidermal growth factor receptor 2.

**Table 4:** Univariable and multivariable Cox regression analysis for overall survival.

Variable	Univariable HR (95% CI)	*p*	Multivariable HR (95% CI)	*p*
Early relapse (≤6 mo)	1.29 (1.00–1.67)	**0.047**	1.14 (0.87–1.49)	0.335
Age ≥ 65 years	0.97 (0.74–1.28)	0.838	—	—
Male sex	1.02 (0.77–1.36)	0.868	—	—
ECOG score ≥ 2	1.46 (0.92–2.32)	0.105	—	—
Grade 3 (poor diff.)	1.43 (1.11–1.85)	**0.006**	1.32 (1.01–1.72)	**0.045**
ypTNM stage III	1.06 (0.81–1.39)	0.669	—	—
Peritoneal metastasis	1.13 (0.87–1.45)	0.354	—	—
Liver metastasis	1.15 (0.87–1.51)	0.327	—	—
HER2 positive	0.57 (0.34–0.94)	**0.028**	0.68 (0.40–1.13)	0.138
Signet ring cell histology	1.50 (1.07–2.10)	**0.018**	1.36 (0.97–1.92)	0.078

Multivariable model includes variables with *p* < 0.10 on univariable analysis: early relapse, grade 3, HER2 positive, and signet ring cell histology. Bold values indicate statistical significance (*p* < 0.05). HR: hazard ratio; CI: confidence interval; ECOG PS: Eastern Cooperative Oncology Group performance status; ypTNM: postoperative pathological tumor-node-metastasis stage; HER2: human epidermal growth factor receptor 2.

## Data Availability

The data that support the findings of this study are available from the corresponding author upon reasonable request. The data are not publicly available due to institutional data protection regulations.

## Abbreviations

The following abbreviations are used in this manuscript:

FLOT	Fluorouracil, Leucovorin, Oxaliplatin, and Docetaxel
GEJ	Gastroesophageal Junction
OS	Overall Survival
PFS	Progression-Free Survival
HR	Hazard Ratio
CI	Confidence Interval
ORR	Objective Response Rate
DCR	Disease Control Rate
MSI	Microsatellite Instability
HER2	Human Epidermal Growth Factor Receptor 2
PD-L1	Programmed Death-Ligand 1
ECOG	Eastern Cooperative Oncology Group
FOLFOX	Folinic Acid, Fluorouracil, and Oxaliplatin
CAPEOX	Capecitabine and Oxaliplatin
FOLFIRI	Folinic Acid, Fluorouracil, and Irinotecan
DCF	Docetaxel, Cisplatin, and Fluorouracil
TOG	Turkish Oncology Group
IO	Immunotherapy
